# Confirmation of double-peaked time distribution of mortality among Asian breast cancer patients in a population-based study

**DOI:** 10.1186/bcr1658

**Published:** 2007-03-06

**Authors:** Fei Gao, Say Beng Tan, David Machin, Nan Soon Wong

**Affiliations:** 1 Division of Clinical Trials and Epidemiological Sciences, National Cancer Centre, 11 Hospital Drive, Singapore 169610; 2 Clinical Trials & Epidemiology Research Unit, Ministry of Health, 226 Outram Road, Singapore 169039; 3 Medical Statistics Group, School of Health and Related Research, University of Sheffield, 30 Regent Street, S1 4DA, Sheffield, UK; 4 Department of Medical Oncology, National Cancer Centre, 11 Hospital Drive, Singapore 169610

## Abstract

**Introduction:**

Double-peaked time distributions of the mortality hazard function have been reported for breast cancer patients from Western populations treated with mastectomy alone. These are thought to reflect accelerated tumour growth at micrometastatic sites mediated by angiogenesis after primary tumour removal as well as tumor dormancy. Similar data are not available for Asian populations. We sought to investigate whether differences exist in the pattern of mortality hazard function between Western breast cancer patients and their Asian counterparts in Singapore, which may suggest underlying differences in tumor biology between the two populations.

**Methods:**

We performed a retrospective cohort study of female unilateral breast cancer patients diagnosed in Singapore between October 1994 and June 1999. Data regarding patient demographics, tumour characteristics and death were available. Overall survival curves were calculated using the Kaplan-Meier method. The hazard rate was calculated as the conditional probability of dying in a time interval, given that the patient was alive at the beginning of the interval. The life table method was used to calculate the yearly hazard rates.

**Results:**

In the 2,105 women identified, 956 patients (45.4%) had mastectomy alone. Demographic characteristics were as follows: 86.5% were Chinese, 45.2% were postmenopausal, 38.9% were hormone receptor positive, 54.6% were node negative and 44.1% had high histological grade. We observed a double-peaked mortality hazard pattern, with a first peak in mortality achieving its maximum between years 2 and 4 after mastectomy, and a second large peak in mortality during year 9. Analyses by subgroups revealed a similar pattern regardless of T stage, or node or menopausal status. This pattern was also noted in high-grade tumors but not in those that were well to moderately differentiated. The double-peaked pattern observed in Singaporean women was quantitatively and qualitatively similar to those reported in Western series.

**Conclusion:**

Our study confirms the existence of a double-peaked process in Asian patients, and it gives further support to the tumour dormancy hypothesis after mastectomy.

## Introduction

The time to event curve describes the cumulative event-free time distribution at a given point after accrual in clinical trials. Although it is widely reported throughout the oncology literature, it does not give direct information regarding changes in event probability over time. Such information is highlighted using the hazard function, which provides the failure rate at any instant among survivors up to that point in time. It is also known as the conditional failure rate or time-specific mortality rate.

Examination of the hazard function in graphical form can provide insight into the pattern of failure. For a treatment that results in cure or prolongation of survival, a decreasing hazard, at least for a period following successful treatment, would be expected [1]. It is therefore somewhat counterintuitive that independent researchers have discovered double-peaked relapse and mortality hazard plots in breast cancer patients treated with mastectomy alone [2-10]. Such double-peaked patterns defy explanation by classical Gompertian tumour kinetics within micrometastatic foci. Neither can they be adequately explained by the existence of distinct patient subpopulations or by the frequency of follow up [2]. One hypothesis suggests that the first recurrence peak corresponds to surgery-induced phase transitions within micrometastatic foci from nondividing single cell states to avascular growth states, and from this state to a phase of vascularization and further growth. The second peak is attributed to activation of micrometastatic foci after several years of tumour dormancy [11].

With increasing recognition that breast cancer is a heterogeneous disease comprising different subtypes with distinct biological behaviours [12], we sought to investigate whether a double-peaked pattern of mortality hazard, similar to that found in Western populations, could be demonstrated in a predominantly Asian cohort.

## Materials and methods

### Data collection

The Singapore Breast Cancer Registry is a prospective cohort of 3,809 females diagnosed with unilateral malignant breast carcinoma from October 1994 to the end of June 1999 in Singapore. All patients registered were prospectively recruited, and their medical records and pathologic reports were retrospectively evaluated.

Biodata including ethnicity (Chinese, Malay, Indian, or other), date of birth, menopausal status and tumor characteristics (oestrogen receptor [ER] and progesterone receptor [PR] status, lymph node status, tumour histological grading, tumour size and pathological T stage [13]) were recorded. Management strategy (type of surgery, adjuvant therapy) was also noted.

Women were considered to be postmenopausal following spontaneous cessation of menses for 6 months or after bilateral oophorectomy. Because the data used are derived from a registry, no detailed information is available on general criteria for adjuvant treatment administration and the methods used to assess hormone receptor status.

Death status was obtained passively from the Department of Cancer Informatics, National Cancer Centre Singapore for patients who received treatment there. For the remaining patients, death status was confirmed by electronic matching with the national death registry. Registration within 2 days is mandatory under Singapore law, and so the completeness of this information can be confirmed as entirely reliable. All women for whom death status was not captured were assumed to be alive at 1 June 2005.

### Statistical analysis

The analysis was focused on mortality. Survival time was calculated from the date of surgery to the date of death or, for those who were alive, it was censored at 1 June 2005. The average interval between date of diagnosis and date of surgery was 11 days. Survival curves were calculated using the Kaplan-Meier product-limit estimator based on deaths from all causes. Hazard ratios, summarizing differences between groups, were estimated using Cox regression and the 95% confidence intervals were obtained.

The annual mortality hazard rate, defined as the conditional probability of dying in a time interval, given that the patient was alive at the beginning of the interval, was calculated as follows [14]: number of women who died within a particular postoperative year/total number of women-years of follow up accumulated in that particular year. Ten-year hazard plots with corresponding 95% confidence intervals were examined for patterns in the changing risk for mortality (Figure 2). To overcome potential masking of underlying mortality hazard patterns using fixed 1-year intervals (bin width), the kernel method of smoothing (Figure 3) with half-width of 2 years was used. Once selected, the deaths within the flexible bin closest in time to the centre of the bin are given more weight in the averaging process. Further successive bins along the time axis overlap, so that a smoothed and continuous estimate of the hazard is obtained [15].

All statistical analysis was completed using the Stata statistical software package (release 7.0; Stata Corporation, College Station, TX, USA), in particular the function sthaz for the kernel plots.

## Results

### Patients

Of 3,809 female patients presenting with unilateral breast cancer, 339 (8.9%) were inoperable, 924 (24.3%) had wide excision, and 2546 (66.8%) underwent total mastectomy. Before mastectomy all patients underwent staging investigations, including liver function test, chest radiography, ultrasound liver, skeletal survey and bone scan.

Among those who underwent mastectomy, 176 patients were excluded because they either had neoadjuvant or palliative treatment (chemotherapy or radiotherapy), and a further 265 patients were excluded because they either had pathological Tis, T0, or T4 disease, or they had stage 4 disease or the stage was unknown. In the remaining 2,105 women (half of whom had received tamoxifen), 45.4% (67.2% of whom received tamoxifen) underwent mastectomy alone, 29.0% received adjuvant chemotherapy without radiotherapy, 6.8% received radiotherapy without chemotherapy and 18.9% received both modalities (Table 1). The mortality hazard patterns among these four groups are examined in detail.

Patients who received adjuvant chemotherapy tended to be younger, whereas the distribution of ethnic descent was similar across the four groups (Table 1). Patients with poor prognostic features such as T2 or T3 disease, positive lymph nodes, higher tumour grade and ER-negative status were more likely to have received adjuvant chemotherapy with or without radiotherapy after mastectomy. Most of the patients who had radiotherapy alone following mastectomy were postmenopausal, and their tumours were larger than 1 cm with moderate to poorly differentiated histological grade.

### Overall survival

**Figure 1:**
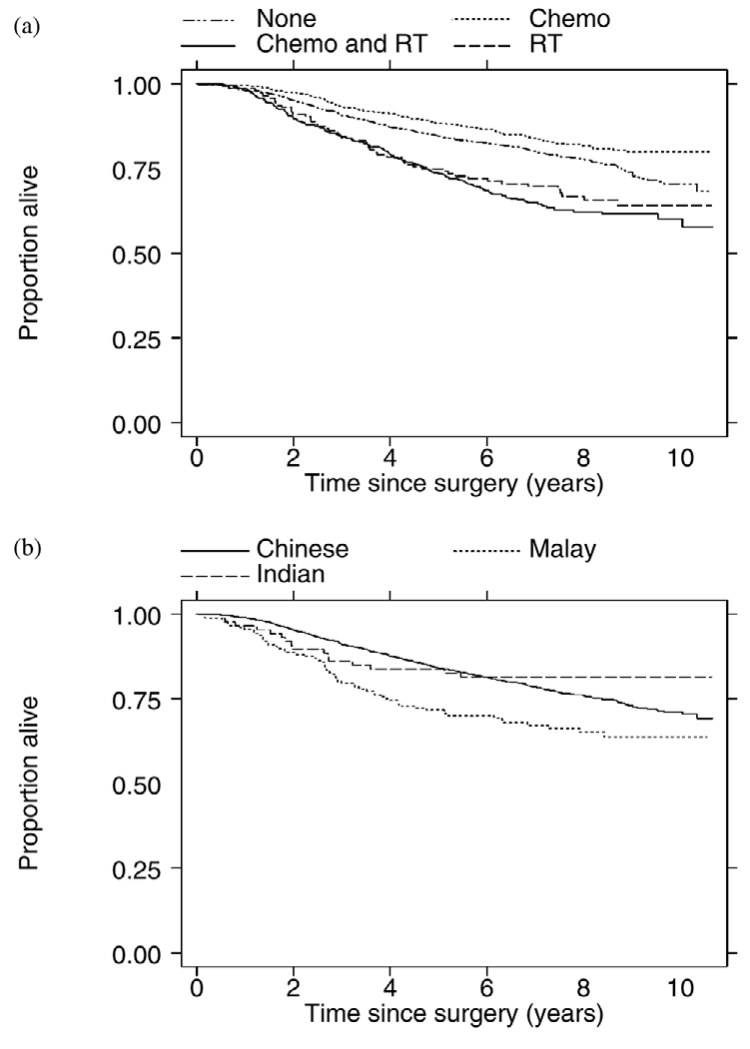
Kaplan-Meier survival estimates. **(a) **Patients undergoing mastectomy alone and those receiving adjuvant chemotherapy or radiotherapy, or both. **(b) **By ethnic group. Chemo, chemotherapy; RT, radiotherapy.

The median follow-up after mastectomy was 7.2 years. As would be expected, the Kaplan-Meier survival curves (Figure 1) show that the poorest survival was in those receiving radiotherapy alone or both radiotherapy and chemotherapy, because these were selected for adjuvant treatment based on poor prognostic features.

Among the major ethnic groups in Singapore, Malay women appear to have the poorest survival (Figure 1). When compared with Chinese, the hazard ratio for these women is 1.59 (95% confidence interval 1.21–2.08), whereas for Indian women it is 0.78 (95% confidence interval 0.48–1.29). After adjustment for type of treatment received, these hazard ratios remain essentially unchanged. There were insufficient patient numbers to adjust further for differences in patient tumour characteristics between ethnic groups.

### Hazard rate

#### Chinese women: mastectomy alone

**Figure 2:**
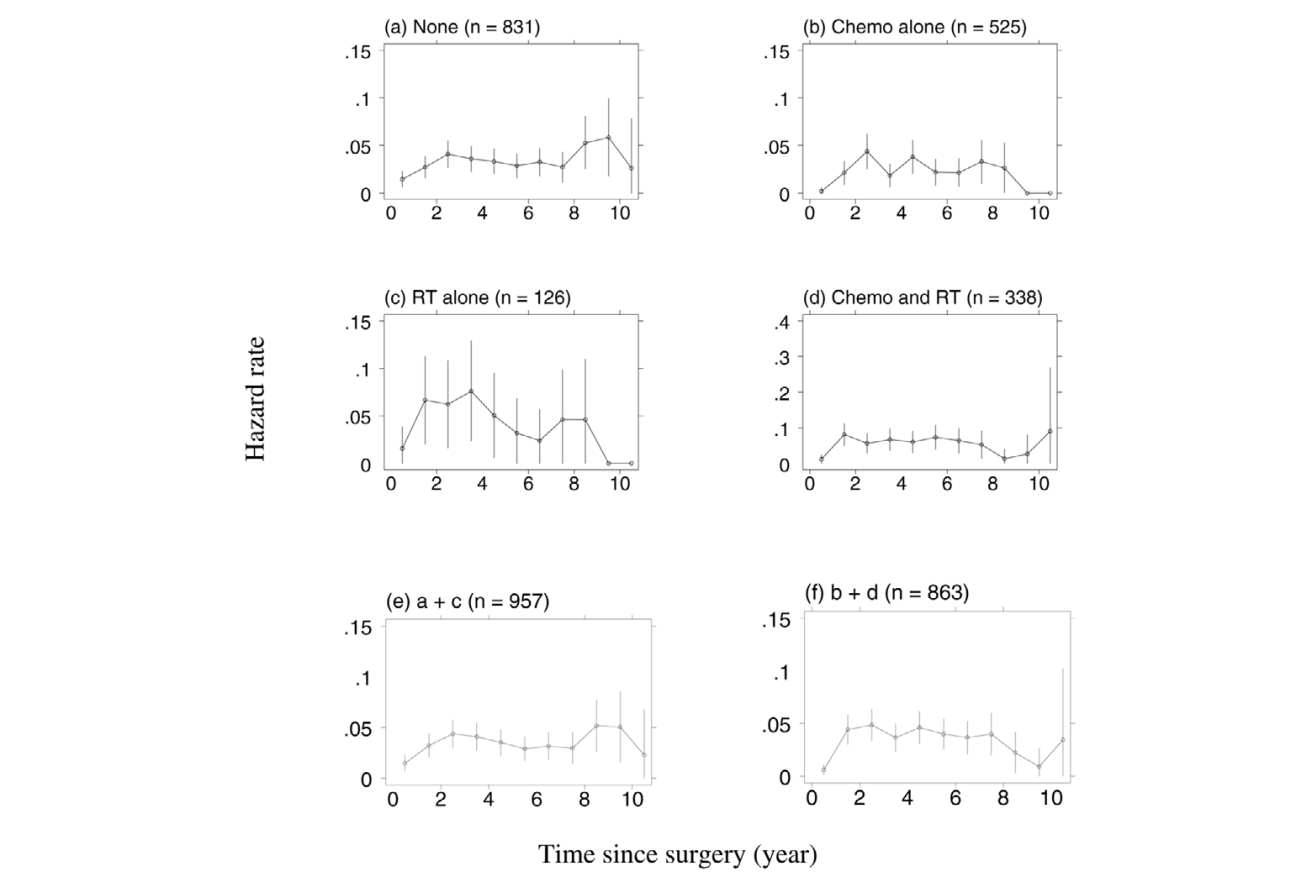
Hazard rates for death in ethnically Chinese breast cancer patients. **(a) **Mastectomy alone. **(b) **Adjuvant chemotherapy without radiotherapy. **(c) **Radiotherapy without chemotherapy. **(d) **Both modalities. **(e) **Local therapy only. **(f) **Systemic therapy. Note the different scale used for the hazard in panel d. Chemo, chemotherapy; RT, radiotherapy.

Because the Chinese comprise the largest ethnic group (80%) in Singapore, and we were interested in comparing our data with those reported by Demicheli and coworkers [5], we first focused on these 831 women. Visual inspection of the annual hazard rates presented in Figure 2a suggests that there is a double-peaked pattern; one peak is rather flat and the second is more pronounced. These are indicated by a gradual increase in risk after surgery until 3 to 4 years, followed by a gradual decline until year 8, and then a second rise to a maximum at 8 to 9 years, although the confidence intervals indicate a lack of precision at each successive time point. However, despite this rather subjective process, the peak times appear to be close to the 3.5 and 8.5 years indicated by Demicheli and coworkers (Figure 1) [5]. Also the mean annual hazard rate of 0.04 is similar to their value of approximately 0.05.

**Figure 3:**
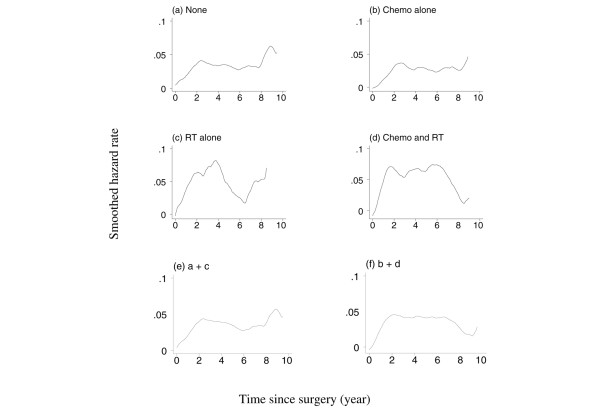
Smoothed hazard for death in ethnically Chinese breast cancer patients. **(a) **Mastectomy alone. **(b) **Adjuvant chemotherapy without radiotherapy. **(c) **Radiotherapy without chemotherapy. **(d) **Both modalities. **(e) **Local therapy only. **(f) **Systemic therapy. Note that the tail of the curve was removed because of the small number of available data. Chemo, chemotherapy; RT, radiotherapy.

The agreement between the peaks identified in our data and those reported by Demicheli and coworkers [5] becomes even more apparent in the corresponding kernel plot of Figure 3a. From this, the estimated time of the first peak is at 2.5 years, with hazard 0.04, and the second is at 9 years, with marginally greater hazard of 0.05. However, with 30.6% (Table 1) of this group comprising women aged 65 years or older, (noncancer) mortality will play an increasing role. Beyond that time point, the follow up data are sparse and so the (smoothed) hazard estimates become unstable.

**Figure 4:**
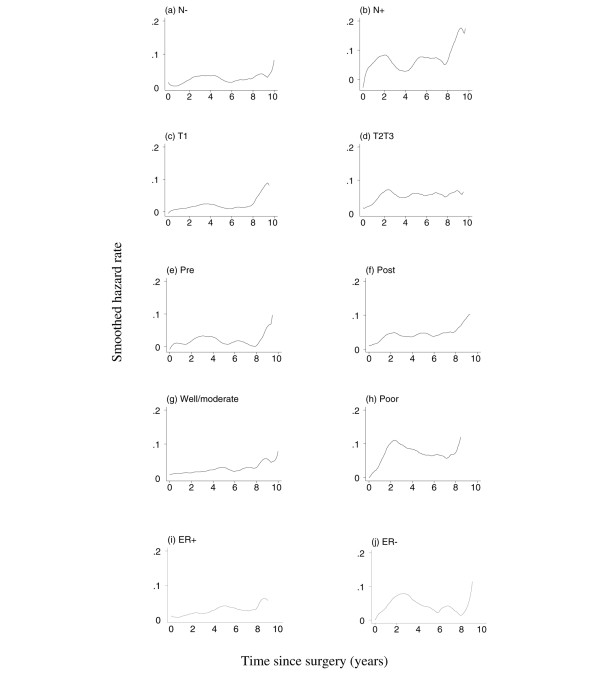
Smoothed hazard for death in ethnically Chinese breast cancer patients having mastectomy alone. **(a) **Lymph node negative. **(b) **Lymph node positive. **(c) **Pathological T1. **(d) **Pathological T2 or T3. **(e) **Premenopausal. **(f) **Postmenopausal patients. **(g) **Well to moderately differentiated. **(h) **Poorly differentiated. **(i) **ER positive. **(j) **ER negative. Note that the tail of the curve was removed because of the small number of available data. ER, oestrogen receptor.

The double-peaked pattern is present in both node-negative and node-positive patients, but the patterns are weak in the former (Figure 4a) and more prominent in the latter (Figure 4b), rising to a maximum hazard at 2 years of 0.1. The mean hazard appears greater, and is relatively uniform over the 4-year to 8-year period, in those whose tumours are T2 or T3 than in those with T1 tumors, among whom there is evidence of a rise after 8 years (Figure 4c,d). There is a discernable but weak double-peak among both premenopausal and postmenopausal women (Figure 4e,f). A distinct peak is evident for poorly differentiated tumours (Figure 4h) but not in well to moderately differentiated tumors.

In these Chinese women treated with mastectomy alone (without chemotherapy or radiotherapy), hormone receptor status was known in 665 (80%) patients, nearly 70% of whom received adjuvant tamoxifen. In those whose ER/PR status was not known, 60% had received tamoxifen. A double-peaked pattern is seen in both ER-positive and ER-negative subgroups (Figure 4i,j). However, somewhat different mortality curves were observed. The first peak for the ER-positive subgroup occurs at 5 years, with a hazard of 0.041, and the second occurs at year 8, with a hazard of 0.058 (Figure 4i). The corresponding peaks for the ER-negative subgroup are at year 3 (hazard 0.072) and year 6 (hazard 0.055; Figure 4j).

#### Chinese women: mastectomy followed by adjuvant treatment

In those patients receiving chemotherapy alone (Figures 2b and 3b) the mortality (mean hazard 0.025) is dissimilar to that in women who underwent mastectomy alone. In contrast, in patients receiving radiotherapy alone (Figures 2c and 3c) the pattern of hazards (mean 0.047) is more variable. Both the chemotherapy alone and radiotherapy alone groups exhibit an early peak at about 2 years, but in the chemotherapy alone group this is followed by gradual decline to a somewhat erratic pattern, fluctuating around a hazard of 0.03. This is in contrast to patients receiving radiotherapy alone, in whom a second and higher peak to a hazard of 0.076 occurs at 3.5 years. However, this group includes only 148 patients (Table 1) and ther are wide 95% confidence intervals for the hazard rates (Figure 2c), and so no pattern is firmly established. In women who received both chemotherapy and radiotherapy, in whom the mortality rises until 1.5 years to an annual hazard of approximately 0.081 and exhibits a plateau at this level until 6.5 years before declining to a minimum at 8.5 years, the double-peak pattern is not apparent (Figures 2d and 3d). As might be expected, the mean hazard for this group, at 0.054, is quite high. Clearly, the double-peak pattern is present in women who underwent local therapy only (the mastectomy alone and radiotherapy alone groups combined; Figures 2e and 3e), and not in those who received systemic chemotherapy (Figures 3f and 3f).

#### Malay and Indian women

**Figure 5:**
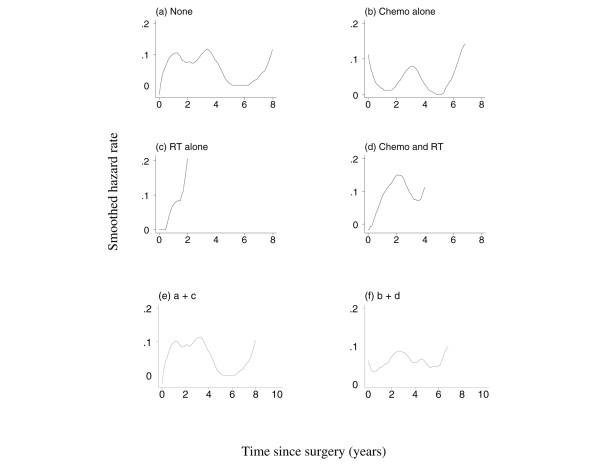
Smoothed hazard for death in ethnically Malay breast cancer patients. **(a) **Mastectomy alone. **(b) **Adjuvant chemotherapy without radiotherapy. **(c) **Radiotherapy without chemotherapy. **(d) **Both modalities. **(e) **Local therapy only. **(f) **Systemic therapy. Note that the tail of the curve was removed because of the small number of available data. Chemo, chemotherapy; RT, radiotherapy.

**Figure 6:**
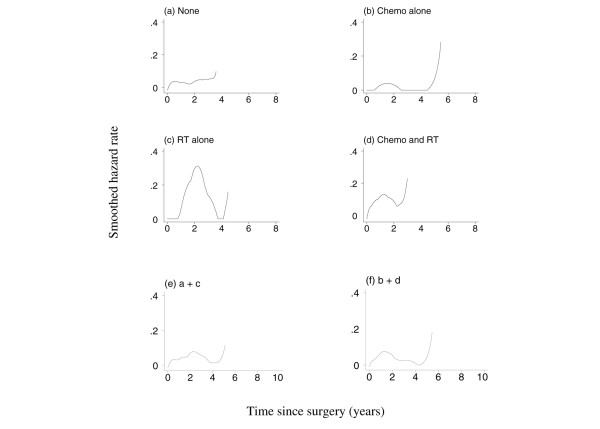
Smoothed hazard for death in ethnically Indian breast cancer patients. **(a) **Mastectomy alone. **(b) **Adjuvant chemotherapy without radiotherapy. **(c) **Radiotherapy without chemotherapy. **(d) **Both modalities. **(e) **Local therapy only. **(f) **Systemic therapy. Note that the tail of the curve was removed because of the small number of available data. Chemo, chemotherapy; RT, radiotherapy.

Because the total numbers of women of Malay (*n *= 176) and Indian (*n *= 86) ethnicity are small, the smoothed hazard plots of the treatment groups are correspondingly less stable and difficult to interpret (Figures 5 and 6). However, there are parallels both between these two ethnic groups and the patterns in the Chinese (Figures 2 and 3), although these can only be speculative.

## Discussion

In the present study, conducted in a predominantly Asian population, we demonstrate the presence of a double-peaked mortality hazard pattern among women treated with mastectomy alone. This double-peaked pattern is observed in the various patient subgroups regardless of hormone receptor status, menopausal status, node status, or T stage; this hints at a true biological phenomenon, as opposed to distinct patient subsets accounting for the separate peaks.

Confining our attention to patients of Chinese origin, we were able to confirm the presence of a double peak but, compared with the data reported by Demicheli and coworkers [5], these peaks occurred at slightly different timings (2.5 and 9.0 years, with corresponding peak hazards of 0.041 and 0.059; Figure 3a). We further verified that double peaks were more prominent in those women with positive nodes and more advanced stage. In addition, although not reported in the Milanese data [5], a double-peak was also found to be present in those with ER-negative tumours.

Our findings are also consistent with those observed in other published reports [5-10] and our study is, to the best of our knowledge, the only one to date that confirms such findings in a non-Caucasian population.

A second important finding was the absence of any double-peaked mortality hazard pattern in women who received adjuvant systemic therapy (with or without radiotherapy; Figures 2f and 3f). In contrast, the double-peak pattern was evident in women who received local therapy (Figures 2e and 3e). This difference may be attributed to the fact that patients in these two groups were biologically distinct, with a larger proportion of women having nodal positivity and higher tumour grade in the combined modality group compared with the mastectomy alone group or radiotherapy alone group. The absence of a double peak in the combined modality arm could also reflect the impact of adjuvant therapy on the natural history of the disease. For example, Demicheli and coworkers [16] found a single peak in the recurrence hazard (which is limited to the first 4 years) in patients with node-positive breast cancer receiving adjuvant treatment (cyclophosphamide, methotrexate and 5-fluorouracil) as compared with a double peak in the control arm (in which patients underwent no further treatment after mastectomy). Saphner and coworkers [17] examined the recurrence hazard of 3,585 women with early breast cancer entered into seven Eastern Cooperative Oncology Group adjuvant trials and demonstrated essentially single-peaked hazard patterns.

It has been shown that mortality risk is associated with menopausal status [5]. In the Milan series, node-positive premenopausal patients exhibited an initial mortality wave covering about 6 years, with a maximum at year 4, followed by a second peak 8 years after surgery; postmenopausal patients, however, exhibited an early high mortality surge peaking at year 3, followed by a modest increase at 8 years. In contrast, we found only a weakly discernable double-peak among the premenopausal and postmenopausal Chinese patients (Figure 4e,f). Part of this may be due to the greater heterogeneity of the Singaporean patients, who were from a population cohort; this contrasts with the Milan series of patients, who were recruited in controlled clinical trials that would have applied carefully defined eligibility criteria.

Patterns among the four broad treatment groups in those of Malay and Indian origin suggest a peak hazard at about 2 years, but this should not be overstated because patient numbers are relatively small in these groups (Table 1, and Figures 5 and 6).

The study has some limitations because we did not have access to recurrence data and were therefore unable to study the association between relapse and mortality hazards. In addition, 70% of women in the mastectomy alone group did receive adjuvant tamoxifen, which is regarded as a form of systemic therapy. However, excluding these patients from the analysis, a double-peak mortality hazard pattern remains (data not shown).

The kernel method of smoothing allows one to conduct a more detailed examination of the changing hazard as compared with the 'annual-bin' method utilized by, for example, Demicheli and coworkers (Figure 1 in that report [5]) and our Figure 2. However, the kernel method of smoothing also involves a degree of subjectivity, so that the relatively minor differences in peak times noted may only reflect chance variations (or differences in statistical methodology) and not systematic differences between Singaporean and Italian women. The smoothed and nonsmoothed estimates become less reliable as postsurgery follow-up time increases and the number of observed deaths declines. The follow up of the Singaporean women continues, and patterns at 10 years after surgery and beyond will be examined in due course.

## Conclusion

In conclusion, we confirm the validity of findings of earlier reports from Milan and elsewhere in some 2,105 South East Asian women, mainly of Chinese ethnicity, who underwent mastectomy for unilateral breast cancer. The risks close to the identified double peaks suggest a hazard that is often double that of the mean hazard for the particular treatment group over time. We therefore provide further evidence for the view presented by Demicheli and coworkers [3] that a double-peak pattern in the hazard function reflects an acceleration in growth of metastases caused by primary tumour removal and activation of dormant sites of micrometastases years after initial treatment. The presence of this phenomenon serves as a reminder of the need for prolonged follow up and surveillance in breast cancer survivors, and suggests that extended or novel antiangiogenic adjuvant therapy may confer benefit in selected patients. Only long-term follow-up research will be able to establish whether newer generation therapies can change the nature history of treated breast cancer and eliminate the second peak.

## Abbreviations

ER = oestrogen receptor; PR = progesterone receptor.

## Competing interests

The authors declare that they have no competing interests.

## Authors' contributions

All authors were involved in the analysis of the study as well as in the preparation of the manuscript. All authors read and approved the final manuscript.

## Figures and Tables

**Table 1:** Characteristics in patients treated with mastectomy by type of adjuvant therapy received

Characteristic	Adjuvant therapy				Total (*n *= 2105)
		
	None (*n *= 956)	Chemotherapy alone(*n *= 610)	Radiotherapy alone (*n *= 143)	Combined chemotherapy and radiotherapy (*n *= 396)	
Age (years)					
<35	14 (1.5)	43 (7.1)	4 (2.8)	24 (6.0)	85 (4.1)
35–49	245 (25.6)	390 (63.9)	32 (22.4)	251 (63.4)	918 (43.6)
50–64	404 (42.3)	161 (26.4)	64 (44.7)	106 (26.8)	735 (34.9)
>64	293 (30.6)	16 (2.6)	43 (30.1)	15 (3.8)	367 (17.4)
Ethnicity					
Chinese	831 (86.9)	525 (86.1)	126 (88.1)	338 (85.4)	1820 (86.5)
Malay	65 (6.8)	62 (10.2)	9 (6.3)	40 (10.1)	176 (8.3)
Indian	46 (4.8)	19 (3.1)	7 (4.9)	14 (3.5)	86 (4.1)
Other	14 (1.5)	4 (0.6)	1 (0.7)	4 (1.0)	23 (1.1)
Menopausal status					
Premenopausal	242 (25.3)	406 (66.6)	35 (24.5)	267 (67.4)	950 (45.1)
Uncertain	91 (9.5)	76 (12.5)	12 (8.4)	25 (6.3)	204 (9.7)
Postmenopausal	623 (65.2)	128 (20.9)	96 (67.1)	104 (26.3)	951 (45.2)
ER/PR status					
+/+	363 (47.1)	176 (32.6)	53 (45.7)	95 (27.9)	687 (38.9)
+/-	139 (18.0)	63 (11.7)	29 (25.0)	61 (17.9)	292 (16.5)
-/+	41 (5.3)	37 (6.8)	2 (1.7)	21 (6.2)	101 (5.7)
-/-	228 (29.6)	264 (48.9)	32 (27.6)	163 (48.0)	687 (38.9)
Not done	185	70	27	56	338
Pathologic T stage					
T1	488 (51.1)	209 (34.3)	38 (26.6)	70 (17.7)	805 (38.2)
T2	423 (44.3)	368 (60.3)	83 (58.0)	249 (62.9)	1123 (53.4)
T3	45 (4.7)	33 (5.4)	22 (15.4)	77 (19.4)	177 (8.4)
Lymph node (N) status					
Positive	243 (25.4)	300 (49.2)	74 (51.8)	339 (85.6)	956 (45.4)
Negative	713 (74.6)	310 (50.8)	69 (48.2)	57 (14.4)	1149 (54.6)
Histological grading					
Well	164 (21.2)	49 (8.8)	17 (13.3)	21 (5.7)	251 (13.8)
Moderate	369 (47.7)	218 (39.2)	57 (44.5)	125 (34.1)	769 (42.2)
Poor	240 (31.1)	289 (52.0)	54 (42.2)	221 (60.2)	804 (44.1)
Not done	183	54	15	29	281

Values are expressed as number (%). ER, oestrogen receptor; PR, progesterone receptor.
